# Nanoantibiotics containing membrane-active human cathelicidin LL-37 or synthetic ceragenins attached to the surface of magnetic nanoparticles as novel and innovative therapeutic tools: current status and potential future applications

**DOI:** 10.1186/s12951-019-0566-z

**Published:** 2020-01-02

**Authors:** Urszula Wnorowska, Krzysztof Fiedoruk, Ewelina Piktel, Suhanya V. Prasad, Magdalena Sulik, Marianna Janion, Tamara Daniluk, Paul B. Savage, Robert Bucki

**Affiliations:** 1 Department of Medical Microbiology and Nanobiomedical Engineering, Medical University of Białystok, Mickiewicza 2c, 15-222 Białystok, Poland; 2 Al. IX Wiekow Kielc 19A, 25-317 Kielce, Poland; 3 Department of Chemistry and Biochemistry, Brigham Young University, Provo, UT 84602 USA

**Keywords:** Nanotechnology, Nanomedicine, Nanoantibiotics, Human cathelicidin, LL-37 peptide, Ceragenins, Membrane active compounds

## Abstract

Nanotechnology-based therapeutic approaches have attracted attention of scientists, in particular due to the special features of nanomaterials, such as adequate biocompatibility, ability to improve therapeutic efficiency of incorporated drugs and to limit their adverse effects. Among a variety of reported nanomaterials for biomedical applications, metal and metal oxide-based nanoparticles offer unique physicochemical properties allowing their use in combination with conventional antimicrobials and as magnetic field-controlled drug delivery nanocarriers. An ever-growing number of studies demonstrate that by combining magnetic nanoparticles with membrane-active, natural human cathelicidin-derived LL-37 peptide, and its synthetic mimics such as ceragenins, innovative nanoagents might be developed. Between others, they demonstrate high clinical potential as antimicrobial, anti-cancer, immunomodulatory and regenerative agents. Due to continuous research, knowledge on pleiotropic character of natural antibacterial peptides and their mimics is growing, and it is justifying to stay that the therapeutic potential of nanosystems containing membrane active compounds has not been exhausted yet.

## Antimicrobial peptides (AMPs) and their synthetic mimics—ceragenins

Antimicrobial peptides (AMPs) are widely distributed in organisms belonging to all branches of the life tree, from invertebrates to vertebrates, fungi and plants to prokaryotic bacteria [1]. Gramicidin, isolated from a soil bacterium *Bacillus brevis* in 1939, is the first characterized antimicrobial peptide, whereas defensin (from rabbit leucocytes), bombinin (from epithelial cells), and lactoferrin (from cow milk) are among the first reported animal-originated AMPs [1].

In microbial ecosystems selective toxicity of AMPs, e.g. bacteriocins, is utilized by microbes in their competitive (antagonistic) interactions [2]. On the other hand, in higher organisms endogenous AMPs are key elements of innate immunity against various microorganisms, including bacteria, fungi, parasites and lipid enveloped viruses [1, 3]. For instance, in animals endogenous AMPs are commonly found on surfaces of mucus membranes and organs directly exposed to pathogens, e.g. skin, airways, intestinal and urinary tract, epithelial cells or lungs, thereby their deficiencies may predispose to skin infections [4], urinary tract infections [5] and periodontal diseases [6].

Currently, over 3500 natural AMPs have been described, ranging in size from five to more than a 100 amino acids [7]. In general, AMPs display significant structural diversity and most of them are classified based on their secondary structure: alpha-helical (e.g., human cathelicidin LL-37), beta-sheet (e.g., human defensins) or peptides with extended/random coil structure (e.g., indolicidin) [3]. Nevertheless, they share overall positive charge and substantial proportion (typically 50%) of hydrophobic residues, thus AMPs are known as cationic antimicrobial peptides (CAPs) [1]. This structural organization, also termed as facially amphiphilic [8], allows them to selectively associate with highly negatively charged microbial membranes and cause defects sufficient for cell death. Their mechanism explains their broad-spectrum of activity, encompassing all cellular pathogens as well as lipid enveloped viruses. Various models of AMP interaction with membranes after initial association through electrostatic forces membranes have been suggested [1, 3]. For instance, the so called ‘carpet model’ is widely accepted, where the membrane is disrupted with formation of micelles as the result of tension exerted by AMPs once their critical concentration on its surface is reached. In addition, AMPs can also interfere with other components of the bacterial cell, such as nucleic acids, regulatory enzymes and other proteins [3].

Beyond direct antimicrobial activity, endogenous AMPs are also effective immunomodulators and thereby indirectly facilitate eradication of pathogens [3, 9, 10]. For instance, AMPs are coupled with (i) inhibition or boosting of inflammation processes through sequestration of bacterial endotoxins and/or by suppression of Toll-like receptors (TLR) and production of proinflammatory cytokines [11], (ii) neovascularization and acceleration of wound healing via stimulation of chemotaxis and cell migration into wound beds [12], (iii) modulation of immune cell differentiation, (iv) initiation of adaptive immunity as well as (v) acceleration bone growth [13]. To illustrate, one of the most extensively studied antimicrobial peptides—human cathelicidin LL-37—inhibits LPS/TLR4-induced secretion of TNF-α in THP-1 cells and suppresses LTA (lipoteichoic acid)/TLR2- and LPS/TLR4-induced production of TNF-α, IL-1β, IL-6 and IL-8 in primary monocytes [14]. Notably, LL-37 is in a phase I/II clinical trial as a topical medication enhancing healing of venous leg ulcers, possibly acting by several mechanisms, including re-epithelialization, angiogenesis, and reduction in inflammation [15].

In spite of the ubiquitous occurrence of endogenous AMPs, pathogens are unlikely to develop high level of resistance to these agents, which makes them good candidates as novel therapeutics. Nevertheless, AMP-resistant strains have been reported and this problem could be an issue with introduction of AMPs to clinical use [16]. Moreover, the peptide nature of AMPs affects their stability and makes them sensitive to destruction by proteases or salt and extreme pH. This observation, in combination with the high cost of peptide therapeutic production and unknown pharmacokinetics, constitute the major obstacles in their broad clinical application.

To overcome these obstacles, ceragenins were developed as non-peptide mimics of AMPs, adopting one of the best studied antimicrobial peptide—polymyxin B as the structural model [8, 17]. The molecular architecture of ceragenins is based on cholic acid, which is appended by amine groups arranged to reproduce the amphiphilic morphology of AMPs. Hence, ceragenins are known also as cationic steroid antimicrobials (CSAs) [18, 19]. This structural similarity allows ceragenins to preserve the broad-spectrum antimicrobial activity of AMPs, but their half-lives are not restricted by the action of proteases, and even long term storage in solutions does not affect antimicrobial capabilities of ceragenins. In addition, ceragenins display significant post-antibiotic effects (PAE), and their smaller size and charge distribution and density, in comparison to AMPs, render them less affected in terms of ability to maintain their bactericidal activity in presence of mucin, DNA or F-actin, which usually accumulates in high concentrations at infected sites [20, 21]. Finally, their synthesis is cost efficient in comparison to relatively complex (~ 20–50 amino acids) AMPs.

Ceragenins are synthetic compounds; however, a related cationic steroid—squalamine, produced by the dogfish shark (*Squalus acanthias*)—has been described [22]. Thus, ceragenins may be classified into two groups: (i) squalamine mimics and (ii) polymyxin B mimics [8]. Squalamine is also potent anti-bacterial, anti-fungal and anti-protozoal agent [22, 23], which seems to be a good argument for further development of ceragenins as mimics of this naturally occurring antimicrobial. At present, ceragenins are promising agents to treat topical infections or in coatings of medical devices. Currently, their usefulness as systemic drugs, alone or in combination with other antimicrobials such as AMPs, classical antibiotics or nanoparticles, is under investigation [24].

## Nanoparticles and nanomaterials as antimicrobial agents

The ever-growing need to design novel antimicrobials with improved pharmacokinetics and with increased killing capabilities against drug-resistant pathogens has directed scientists’ interest in nanotechnology and nanomedicine. Currently, use of nanomaterials is an attractive therapeutic approach for the development of novel antimicrobials in the era of constantly growing antibiotic resistance.

Nanoparticles (NPs) are nano-sized molecules (usually < 100 nm in diameter), composed of (i) metals, (ii) lipids (e.g., liposomes), (iii) natural antibacterial substances (e.g., chitosan), (iv) biodegradable polymers (e.g., polylactic acid), (v) carbon-based polymers (e.g., fullerenes) as well as (vi) surfactant-based nanoemulsions (e.g., soybean oil, Tween 80) and (vii) dendrimers [25–28]. Owing to their unique physicochemical properties NPs have become of great interest in medicine over the last decade. Presently, Au, Ag, Cu, Zn, Ni, Ti, Mn, Al, Si, Fe, Cl, Bi and Ir are commonly used to synthesize nanoparticles [27, 29]. Metal-based nanoparticles commonly show unique electrical, magnetic, thermal, dielectric or optical potential, which can be used to target (e.g., by magnetic guidance or radio frequency) or to activate NPs in the site of action (e.g., by X-rays, UV light or laser irradiation) [26, 30]. Notably, a large surface area to volume ratio (SA:V) of NPs, facilitates their strong interactions with microbial membranes, exerting antimicrobial activity even in minute doses, as well allowing efficient surface functionalization [31, 32]. Hence, NPs have emerged as alternatives for antimicrobial agents and drug delivery systems [28, 33–35]. Furthermore, the biological activity of NPs depends on various characteristics, including size, shape (spherical, triangular, rod, cube, square, flake, plate, ellipsoidal, irregular, etc.), surface charge, type of material and concentration [29]. In general, the smaller the nanoparticle the better antimicrobial activity; however, size alone is not the most important determinant of their activity [29]. Shape also plays a role; for instance, rod shaped NPs may be more effective in bacterial biofilm destruction than spherical examples [36].

There is a compelling amount of evidence indicating that magnetic nanoagents may display a variety advantages over the traditional antibiotics. For example, they were reported to be (i) less susceptible to bacterial resistance and (ii) to be amenable to functionalization to optimize activity against preferred targets [37]. Moreover, nanoparticles-based agents might be stimulated in various ways, including temperature, pH or magnetic fields [38]; in addition, they are resistant to the biodegradation processes and possess the ability to penetrate bacterial cell membranes and cross some barriers that are typically non-permeable for conventional therapeutic agents [39]. Importantly, association of an antibiotic with a NP may display a cumulative effect on pathogenic microorganisms and thus overcome the resistance mechanisms and lower effective drug doses thereby reducing undesirable side effects [40]. In addition, the therapeutic efficacy of traditional antimicrobials may be further improved by aggregation of multiple antimicrobials within one NP or conjugation of NPs with pathogen-specific antibodies (e.g., anti-peptidoglycan or anti-protein A *Staphylococcus aureus* antibodies [30]) or ligands (e.g., mannose to kill intracellular bacteria expressing mannose-receptors in alveolar macrophages [41]) for targeted delivery. Furthermore, NPs such as liposomes and dendrimers (hyperbranched polymers) may bypass resistance mechanisms associated with (i) decreased uptake or increased efflux of drugs from the bacterial cell, (i) biofilm formation, as well as intracellular localization of bacteria [40]. Finally, NPs can improve pharmacokinetics of poorly water-soluble drugs, prolong their half-life and systemic circulation time [26]. Therefore, NPs alone or in combination with traditional antimicrobials are highly promising tools in fighting existing multidrug-resistant microbes and in preventing their emergence in the future.

There are also concerns associated with using NPs as therapeutics. Although their remarkable stability could be beneficial from the therapeutic point of view, associated side effects include the potential risk of their deposition in various organs (and in the environment), particularly upon long-term exposure, posing a serious problem that may limit their application [25, 26, 33, 42]. Other negative effects must also be considered, including cytotoxicity and induction of apoptosis, as well as genotoxic and carcinogenic actions [25]. Some NPs such as Al_2_O_3_ may increase antibiotic resistance by promoting horizontal gene transfer of MDR genes, [43] in addition to acquired resistance that has been reported for silver NPs [44].

Although mechanisms of action of NPs on microbial cells are not fully understood, similar to AMPs and ceragenins, the cell membrane appears to be their primary target. Membrane damage of a physicochemical nature, with a “hole” or “pore” formation, occurs as the result of non-specific electrostatic binding of NPs and membrane insertion [45]. In addition, generation of reactive oxygen species (ROS), release of free metal ions as well as interactions with intracellular components such as DNA, ATP and proteins, are possible modes of action [28, 29, 34, 35, 40]. For example, it has been demonstrated that Cd^2+^ and Zn^2+^ can bind to sulfur-containing proteins of the cell membrane and interfere in cell permeability, and cell respiration is inhibited when chloride ions precipitate as silver chloride in the cytoplasm of the cells. Ag^+^ ions can also damage DNA by inhibiting its replication [15]. Since metal- or chitosan-containing NPs, and nitric oxide-releasing (NO NPs) utilize multiple mechanisms simultaneously to kill microbial cells, the emergence of resistance to these molecules is unlikely. Interestingly, like AMPs and CSAs, NPs may support clearance of pathogens indirectly by triggering innate and adaptive immune responses [33].

## Antimicrobial activity of nanosystems functionalized by LL-37 and ceragenin CSA-13

Among a variety of nanomaterials evaluated for the purposes of antifungal and antibacterial therapies, nanostructures based on iron oxide demonstrate promising applications. To date, strong antimicrobial activity of bare iron oxide magnetic nanoparticles (MNPs) against a broad spectrum of bacteria, including *Escherichia coli, Pseudomonas aeruginosa, Serratia marcescens, Staphylococcus aures, Bacillus subtilis* and *Proteus mirabilis* [46, 47], both in vegetative form and, importantly, also against biofilm developed by these pathogens [48], was reported. Their therapeutic potential is also further highlighted by the fact that they can be functionalized with a broad range of antimicrobials, including conventional antibiotics (e.g., amoxicillin [49], amphotericin B, nystatin [50], ciprofloxacin [51] and disinfectants [52]). Newer studies propose the engagement of iron oxide-based nanoparticles in combination with endogenous antimicrobial peptides, particularly human cathelicidin-derived LL-37 peptide and its mimics, ceragenins.

Magnetic nanoparticles were reported to be highly valuable nanocarriers of AMPs, increasing therapeutic effectiveness of these agents while simultaneously decreasing adverse effects such as hemolysis [53]. In effect, AMPs-based nanoformulations seem to fit in the current trend of developing novel antibiotics with alternative mechanisms of action, broad spectrum of antimicrobial activity, low tendency to induce drug resistance, with appropriate bioavailability and reasonable synthetic costs.

### Ceragenins as potent and highly effective antimicrobials

Cathelicidin LL-37 and its mimics, including ceragenins, are characterized by a broad spectrum of activity against a variety of microbial pathogens, including both Gram-positive and Gram-negative bacteria and multidrug-resistant strains [54–60]. Importantly, the tendency of drug resistance induction is considerably lower for this class of compounds when compared to conventional antibiotics; it is known that ceragenin CSA-13, being the best described and characterized agent of this group, retain its potent antibacterial activity over the course of 30 serial passages, indicating that bacteria are very unlikely to develop resistance toward this agent [61].

The mechanism of bactericidal action proposed for these compounds involves electrostatic association between LL-37/ceragenins and the negatively charged bacterial membranes [3, 62], which allows for further direct and rapid antimicrobial activity associated with microbial membrane insertion, leading to changes in membrane phospholipid organization and rapid membrane depolarization [63]. Notably, it has been shown that AMPs and their non-peptide mimics may damage mammalian cell membranes due to their membrane-permeabilizing capabilities, but this effect is observed at concentrations higher than that required to obtain bactericidal effects and restrict pathogen growth [64].

**Fig. 1:**
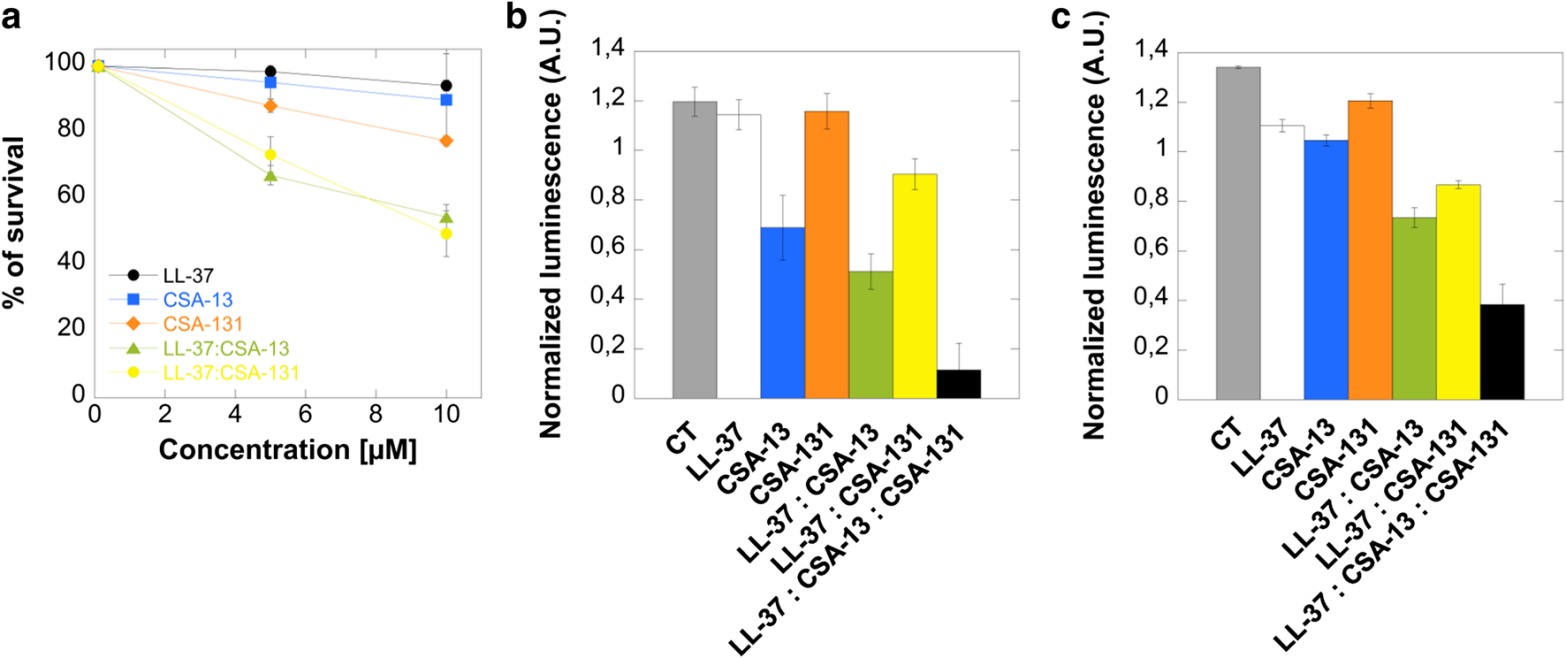
Antimicrobial activitity of LL-37 peptide and ceragenins CSA-13 and CSA-131 both alone and in combinations against extracellular and intracellular pathogens. **a** Intracellular killing efficacies of LL-37 (black circles), CSA-13 (blue squares), CSA-131 (orange diamonds), and combination of LL-37 with CSA-13 (green triangles) and LL-37 with CSA-131 (yellow circles) against *Candida albicans* within bladder epithelial cells. The concentrations of used antibacterial agents were 5 and 10 μM; incubation time was 2 h. **b**, **c** Reduction of *P. aeruginosa* Xen 5 (grey column) chemiluminescence signal (~ 10^8^ CFU/ml) after LL-37 (10 µg/mL; white column); CSA-13 (10 µg/mL; blue column); CSA-131 (10 µg/mL; orange column) and combination of LL-37 with CSA-13 (1:1 10 µg/mL each; green column); LL-37 with CSA-131 (1:1 10 µg/mL each; yellow column); and LL-37 with CSA-13 with CSA-131 (1:1:1 10 µg/mL each; black column) after 30 min of incubation in LB broth (**b**) and urine (**c**)

The therapeutic usefulness of ceragenins has been highlighted by multiple publications, suggesting that CSA-13 displays stronger bactericidal activity than LL-37 not only for aerobic bacteria but also for anaerobic pathogens, including *Bacteroides spp*. and *Clostridium difficile* [65, 66]. Recent data also showed the efficiency of CSA-13 against carbapenem-resistant *Acinetobacter baumannii* and highly resistant *Pseudomonas aeruginosa* isolates and against the hypervirulent strain LESB58 even at low concentrations [56, 67]. Particularly, studies performed in airways mimicking environmental exposure expanded the knowledge about the antibacterial potential of CSA-13. Apart from the potent anti-*P. aeruginosa* LESB58 action, CSA-13 was characterized by more favorable features than LL-37 peptide that is naturally present in airway surface fluid. Most importantly, in contrast to the human cathelicidin, the activities of CSA-13 were not affected in the presence of DNA, polymerized actin and Pf1 bacteriophages that are highly and naturally expressed by *P. aeruginosa* LESB58. These properties support the hypothesis that ceragenins are suitable for developing effective treatment against *P. aeruginosa* lung infections in cystic fibrosis patients [20, 56]. Other published studies investigated the high bactericidal activity of CSA-13 against *Staphylococcus aureus*, including vancomycin-resistant strains or using erythromycin-ceragenin combinations as a new approach to eradicate MDR pathogens [68]. MIC values were compared with various conventional antibiotics, and overall CSA-13 was the most effective with an MIC range 1–2 µg/ml [69]. A recent study indicated that the spectrum of antimicrobial activity of ceragenins includes *Legionella pneumophila*, using CSA-8, CSA-13, CSA-44, CSA-131 and CSA-138 [70]. Ceragenins were also recorded to be highly effective against *Bacillus subtilis* spores [71]. Interestingly, CSA-13 possessed greater affinity for the spores than for the vegetative form of bacteria which is caused by more negative surface charge (− 26 mV) in spores than vegetative cells (− 21 mV), which is in agreement with the proposed CSA-13 mode of action involving electrostatic interactions [71]. In addition to the reports described above, some of our data [72] suggest the therapeutic value of CSA-13, CSA-131 and their combinations with LL-37 peptide in the eradication of *E. coli*, isolated from patients diagnosed with recurrent urinary tract infections. Particularly, ceragenin CSA-131 exerts strong synergistic effect with LL-37, which supports the hypothesis about the reinforcement of host natural defense by application of cationic lipids (Tables 1, 2 and Fig. 1) [72].

**Table 1:** Minimal inhibitory concentrations (MIC; μg/mL) of tested agents against clinical strains of *Escherichia coli* from patients diagnosed with recurrent urinary tract infections

Strains	AMP	LL-37	CSA-13	CSA-131	LL-37 + AMP	LL-37 + CSA-13	LL-37 + CSA-131
*E. coli*	4	64	2	16	4	2	1
*E. coli*	4	64	4	8	4	2	2
*E. coli*	4	64	4	16	4	2	2
*E. coli*	4	32	4	16	4	0.25	2
*E. coli*	4	64	2	16	4	2	2

AMP, ampicillin; *E. coli*, clinical isolate of *Escherichia coli*

**Table 2:** Fractional inhibitory concentrations indices (FIC index) of combination of LL-37 with CSA-13 and CSA-131 against clinical strains of *Escherichia coli* from patients diagnosed with recurrent urinary tract infections

Strains	LL-37 + CSA-13 FIC index/interpretation	LL-37 + CSA-131 FIC index/interpretation
*E. coli*	1.03125/AN	0.078125/S
*E. coli*	0.53125/PS	0.25125/S
*E. coli*	0.53125/PS	0.15625/S
*E. coli*	0.0703125/S	0.1875/S
*E. coli*	1.03125/AN	0.15625/S

FIC values > 4 indicate antagonistic effect (AN); values between 4 and 1.01 indicate indifference (I); values between 1 and 0.76 indicate additive effects (AD); values < 0.76–0.5 indicate partial synergy (PS) and < 0.5 indicate synergistic effect (S)

AMP, ampicillin; *E. coli*, clinical isolate of *Escherichia coli*

Results from in vitro studies were expanded upon with in vivo studies aiming to evaluate the therapeutic efficiency, but most importantly, safety of potential treatment with ceragenins. To date, strong antibacterial and anti-inflammatory activities of CSA-13 were observed in a mouse model of *Pseudomonas aeruginosa* infection [59]. Results obtained in an animals with peritonitis after direct administration of bacteria into the abdominal cavity, suggest the strong potential of CSA-13 for future development as a new class of antibiotic. It is postulated that the in vivo activity of CSA-13 probably depends on environmental factors at the site of infection, the host’s immune response and the type of infection caused by the pathogen [59, 73]. Importantly, in vivo studies of CSA-13 show low toxicity, supporting the possible applications of ceragenins in human treatment [68]. Pharmacokinetic features of ceragenin CSA-13 are comparable to those of conventional antibiotics and similar to fluoroquinolones [74].

Recently published data suggest some intracellular activity of ceragenins (CSA-13, CSA-131) that may be particularly valuable in the therapy of highly recurrent infections, such as urinary tract infections (UTIs). Currently, the effective treatment of UTIs is complicated by the presence of intracellular pathogens, which are internalized by infected host cells and thus, are able to avoid killing by antibiotics and natural antimicrobial compounds. Considering that a majority of currently used antimicrobial agents is ineffective against intracellular forms of pathogens, the development of novel ceragenins with ability to penetrate the cells and to exert bactericidal effects, is of great interest.

### Nanotechnology-based drugs containing LL-37 and ceragenins to prevent microbial infections

Many recent studies show that nanotechnology offers new tools for developing therapies to effectively kill microbial agents, including those characterized by antibiotic resistance [35, 52, 75]. Among these, there are reports of immobilization of ceragenins on the surface of nanomaterials and activities of such nanosystems against bacteria and cancer cells. One study indicates that antimicrobial features of silver, which is characterized by broad-spectrum antimicrobial properties, might be additionally complemented by conjugation with selective ligands such as CSA-124. Silver NPs coated with ceragenins, or other cationic antimicrobials, were found to be eight times more effective against bacteria than silver NP alone. Interestingly, despite the potent killing abilities of CSA-124-conjugated NPs, activity of silver NPs against both *S. aureus* and *E. coli* with some selectivity against *S. aureus* over *E. coli* were observed. The mechanism of this phenomenon invites further investigation [76].

Series of experiments indicate the possibility to successfully employ the iron oxide-based magnetic NPs as nanocarriers for ceragenins. Comparison of bactericidal activity of free ceragenin CSA-13 and its magnetic derivative composed of CSA-13 attached to the surface of aminosilane-coated magnetic NP (MNP@CSA-13) performed by Niemirowicz et al. [77] demonstrated that the nanocomposite is more efficient in killing of *P. aeruginosa* than CSA-13 alone. Notably, the study described above indicated that attachment of ceragenin CSA-13 to a NP not only significantly improved its bactericidal and anti-biofilm properties in the PBS-based environment, but also improves bactericidal activity in the presence of bodily fluids (such as urine, saliva, plasma, pus, ascites, cerebrospinal fluid, bronchoalveolar lavage and cystic fibrosis sputum) suggesting that MNP@CSA-13 might be used as nanocarrier for controlled antibiotic delivery at infection sites [77]. Controlled release of CSA-13 from MNPs may allow targeted delivery of the antimicrobial; since lower pH values were reported to accelerate imine bond hydrolysis, resulting in liberation of CSA-13 from nanoparticles, it might be successfully used in in the eradication of *Helicobacter pylori* [77] (CSA-13 displays strong bactericidal activity against *H. pylori* [57]). Likewise, attachment of LL-37 or CSA-13 and CSA-131 to NPs revealed comparable or stronger bactericidal activity against common anaerobic bacterial pathogens [65], as well as increased ability to prevent biofilm formation by *Bacteroides fragilis* and *Propionibacterium acnes* [65]. Another study highlighted the use of magnetic nanoparticles to exert additive and synergistic activity with LL-37, ceragenins CSA-13 or CSA-131 and conventional antibiotics (i.e. vancomycin and colistin) against methicillin-resistant *S. aureus* (MRSA) and *P. aeruginosa* through formation of electrostatic interactions [37]. This observation is consistent with previous reports demonstrating MNP-mediated modulation of bactericidal activity of antibiotics from different groups, which is likely caused by increasing membrane fluidity of targeted organisms [50, 78]. The considerable increase of antimicrobial activity of these antibiotics in the presence of MNPs may (i) increase their therapeutic value against drug resistant strains and/or (ii) decrease active doses, and thereby limit adverse effects. Importantly, such preparation of CSA-based nanoformulations, using electrostatic instead of covalent attachment of agents to nanoparticles might significantly reduce the production costs of NP-associated materials, in comparison to their covalently attached counterparts.

A recent publication describes incorporation of a ceragenin into multifunctional, spherical nanosystems with iron oxide cores, reduced silver shells with a ceragenin CSA-124 (thiol-containing ceragenins) monolayer. This nanosystem was shown to adhere to *S. aureus* in vitro, and its use was proposed as a diagnostic agents for identifying deep tissue infections as a T2 MRI negative contrast agent [79]. This use highlights activities of ceragenins that might be valuable in the design of theranostic nanoconjugates for diagnostic purposes.

A key aspect that must be considered in the design of novel antimicrobials is their safety and biocompatibility. Compelling evidence suggests that magnetic nanoparticles display low toxicity and biodegradability [80], which allow for their employment as non-toxic drug carriers. On the other hand, AMPs at high concentrations might exert some toxic effects to hosts cells as the consequence of their membrane activity [81]. Similarly, CSA-13’s ability to interrupt membrane lipid architecture in bacterial cells is not entirely selective, and thus, at higher concentrations, CSA-13 might affect the lipid organization within host cell membranes, resulting in hemolysis of red blood cells [82]. Notably, this effect is observed in doses that exceed the effective bactericidal concentrations. Nevertheless, to address possible toxicity, combinations of various antimicrobials, including cathelicidin LL-37 and ceragenins with core–shell magnetic NPs were reported, and these not only increased killing capabilities against bacteria, but also significantly increased biocompatibility of such combinations [37, 77]. Decoration of silver and iron nanoparticles by ceragenins CSA-124 and CSA-13 by both non-specific silver-sulfur bonding or covalent bonding, respectively, was found to decrease significantly release of hemoglobin from erythrocytes, even at relatively high concentrations [77, 83]. Interestingly, immobilization of ceragenins into electrostatic-based NPs might more effectively improve the hemocompatibility of CSA-based nanosystems when comparing to chemical adsorbed nanoagents (unpublished data). The attachment of CSA-13 to the surface of iron oxide nanoparticles also decreased toxic effects against a human osteoblast cell line at high concentrations [84].

### Antifungal activity of ceragenins and CSA-based nanoformulations

Emerging drug-resistant fungal strains necessitates the development of novel antimicrobial therapies. Despite a high number and diverse group of currently available antibacterial drugs, the number of active substances for treatment of pathogenic fungi is still limited [85]. In order to overcome the resistance of pathogenic fungi, often found in polymicrobial infections, various therapeutic approaches have been used, including modifications of the chemical structure of known antifungals, use of drug transporters and combined drug therapies. Application of combination therapy is associated with decreased dose-related toxicity as a result of reduced dosages and antifungal synergy [37, 50, 86]. An attractive class of combination therapy includes membrane-permeabilizing agents and nanomaterials as drug carriers [84].

To date, several reports demonstrate antifungal features of magnetic nanoparticles, and this activity is due to (i) disruption of membranes, (ii) electrostatic and hydrophobic interaction with proteins on fungal cells, (iii) induction of fungal cells apoptosis and (iv) impairment in ergosterol signalling and efflux pump functions [87]. Mechanisms of action of antifungal AMPs are attributed to (i) promotion of damage by reactive oxygen species; (ii) attenuation of mitochondrial functions leading to apoptosis and (iii) membrane perturbation [88–92]. The latter effect is proposed to predominate with ceragenins, considering the amphiphilic morphology of both AMPs and ceragenins. Indeed, membrane-permeabilizing properties of LL-37 and ceragenins against fungi was confirmed by atomic force microscopy (AFM)-based measurements, which showed changes in surface morphology of *Candida albicans* treated with LL-37 and CSA-13 with small, crack-like breaks in the cell surfaces and increased surface wrinkling for LL-37- and CSA-13-treated samples, respectively [86]. The analysis of differences in lateral deflection images might potentially indicate two different, antifungal mechanisms of these agents.

Multiple reports describe the antifungal activities of ceragenins and ceragenin-based nanosystems against both laboratory and clinical fungal strains. Most importantly, these antifungal activities extended to drug-resistant strains. To date, CSA-13, CSA-44, CSA-131, and CSA-138 were reported to be highly effective in killing of 50 *C. albicans* planktonic and biofilm-embedded fungal strains, both alone or in combination. As reported, synergistic interactions were variable between compounds with CSA-13 exerting the strongest synergistic effect with amphotericin B, and CSA-131 exerting the weakest interaction [93]. The antifungal activity of CSA-44, CSA-131, CSA-142 against 100 clinical isolates of *C. auris*, showed additionally that ceragenins were active at very low concentrations (0.5 to 2.0 mg/L) against all tested strains, including those that were fluconazole-resistant and echinocandin-resistant [94]. Research reported by Durnaś et al. demonstrated that clinical and environmental isolates of yeast or filamentous fungi from *Candida, Cryptococcus, Aspergillus, Scedosporium, Rhizopus*, *Blastomyces* and *Apophysomyces* species were also susceptible to the ceragenins [86]. Notably, ceragenins were able to maintain activity in the presence of body fluids, in which various factors, including proteases, are responsible for limitation of LL-37 activity [95].

The unique properties of magnetic nanoparticles allow for their functionalization with antifungal antibiotics, including ceragenins, enabling the optimization of the fungicidal effect [84]. To date, antifungal activity of LL-37 peptide, CSA-13 and its magnetic derivatives (MNP@LL-37, MNP@CSA-13) against laboratory and clinical strains of *C. albicans*, *C. glabrata* and *C. tropicalis* were assessed [84, 86]. In analogy, to unmodified ceragenins, high anti-fungal activity of tested compounds was demonstrated to be mediated by their interaction with the fungal membrane. Simultaneously, enhancement of the fungicidal activity of MNP@LL-37 and MNP@CSA-13 was determined by the ability of nanosystems to penetrate cell membranes and growing cellular uptake of antifungal agents. Moreover, magnetic nanoparticles were noted to trigger oxidative damage of *Candida* cells, which results with organella disfunction and leads ultimately to cell death without the affecting considerably host cells proliferation and cytokine profile. Accordingly, in fungal cells treated with ceragenin-functionalized nanoparticles ROS level was significantly higher than in fungal cells incubated in the presence of non-magnetic counterparts [84]. Nevertheless, additional experiments are still obligatory to fully understand how CSAs target and kill fungal cells.

### Antibiofilm activity

The effective eradication of microbial infections is complicated further by the development of biofilms, which is recognized as a primary factor leading to increased resistance to antibiotic treatment and survival of bacteria. Therefore, development of new strategies for combating bacterial biofilm has become a high priority. AMPs, ceragenins and their magnetic derivatives are characterized by a broad spectrum of killing capabilities, including the ability to inhibit adhesion and formation of mature biofilms and disruption of established biofilms. Thus, these agents are potential candidates to treat not only acute infections caused by planktonic bacteria but also biofilm-associated infections.

Reported studies demonstrate that LL-37, ceragenins, MNP@LL-37 and MNP@CSA-13 inhibit the formation of biofilms by both bacteria and fungi. Data reported by Nagant et al. demonstrate that CSA-13 exerts high antibacterial activity against pre-formed biofilms of *P. aeruginosa*; more than half of *P. aeruginosa* biofilm bacteria were killed at 50 mg/L and 100% at 100 mg/L [96]. Low concertations of CSA-13 were reported to inhibit the *P. aeruginosa* biofilm formation through electrostatic interactions and without affecting the production of rhamnolipids [97]. Furthermore, CSA-13 also limits the adhesion of *P. aeruginosa* to an abiotic surfaces and is bactericidal on cells embedded within mature biofilm, which confirmed the therapeutic value of this compound against all stages of biofilm formation [97, 98]. Studies performed by our research team show that the cationic lipids are able to prevent *P. aeruginosa* LESB58 biofilm formation more effectively than LL-37 peptide [56]. Moreover, the antibiofilm activity of CSA-13 was maintained in the presence of negatively-charged polyelectrolytes released from injured host cells (i.e. F-actin, DNA) or produced by bacteria (bacteriophage Pf1), each of which showed a strong ability to induce biofilm formation [56]. In addition to these reports, additional papers described the activities of a few ceragenins, including CSA-8, CSA-13, CSA-44, CSA-131, and CSA-138 in inhibiting the formation of biofilms (24 h and 48 h), in a concentration-dependent manner [82, 93, 96].

Bare magnetic nanoparticles, due to their unique properties including superparamagnetism, offer the possibility of use as (i) enhancers of anti-biofilm activity of unmodified antibiotics or (ii) as sensitizing tools in modern photodynamic therapy or magnetic fluid hyperthermia-based anti-biofilm treatments. Studies indicate that antimicrobial agents attached to MNPs (MNP@LL-37, MNP@CSA-13), and even MNPs alone, exhibit potent/enhanced antibiofilm activity [65]. Importantly, low doses of nanosystems are required to effectively inhibit biofilm formation, while five or ten times higher concentrations of the free, nonmodified drug were required to obtain the same effect [50]. Ceragenin-based nanosystems were reported to be highly effective in eradication of mature *P. aeruginosa* biofilms [77]; similar effect was also noted for biofilm formed by *S. aureus* MRSA [37]. The ability of the LL-37, CSA-13 and MNP@LL-37, MNP@CSA-13 to modulate fungal adhesion and biofilm formation was assessed by Niemirowicz et al. [84]. In this study, *Candida albicans* cell adhesion was reduced by 40% in the presence of MNP@ CSA-13 as compared to CSA-13 alone. With LL-37 immobilization on the surface of MNPs, a three-fold lower rate of adhesion of *Candida tropicalis* cells was also noted [84]. Considering the previous reports indicating strong antibiofilm activities of unmodified metal nanoparticles against *C. albicans* biofilms (determined by cell wall disruption followed by loss of fungal biofilm structure [99]), it is suggested that improved antibiofilm features of LL37/ceragenin-based nanosystems results from combined membrane-permeabilizing properties of ceragenin and nanomaterials.

## The employment of membrane active compounds-containing nanosystems for the modern therapy of cancer

**Fig. 2:**
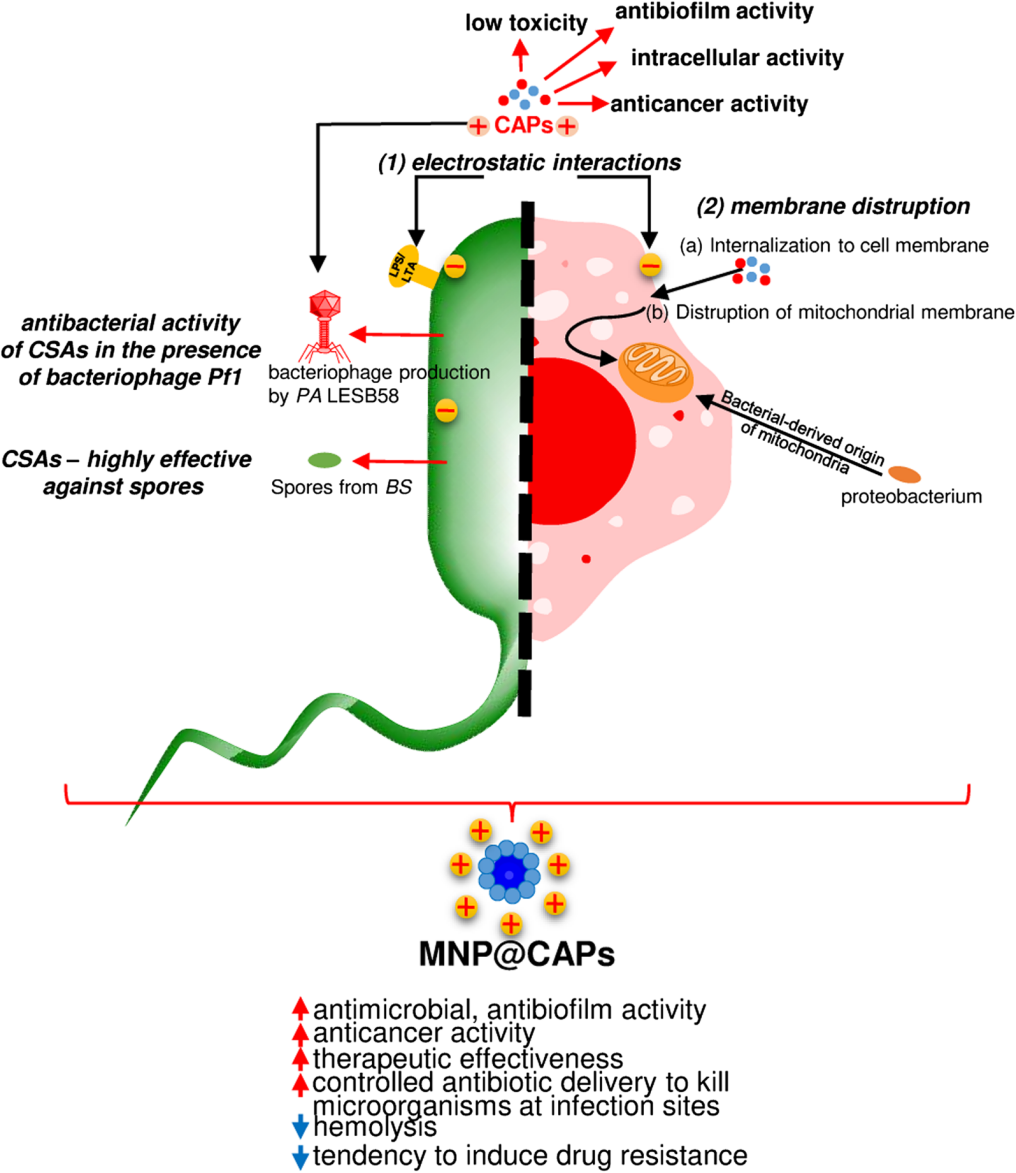
Broad spectrum of activities reported for cationic antimicrobial peptides and CAPs-containing nanosystems

Despite the unprecedented achievements in the field of modern anti-cancer therapies, a majority of chemotherapeutics lack specificity to transformed cancer cells and thus, exert toxic effects against healthy, highly proliferating cells [100]. Simultaneously, the development of resistance by tumor cells in the response to chemotherapy is also recognized as a considerable limitation governing modern anti-neoplastic therapies [101]. Consequently, more effective therapeutic alternatives and novel mechanism-based approaches are needed to (i) target cancer cells without displaying toxicity to normal cells undergoing rapid proliferation and (ii) decrease the propensity to induce drug resistance. Studies on bacterial cells are the basis for examination of membrane-active agents as components of modern anti-cancer therapy. Although AMPs and ceragenins have been studied primarily as potent anti-infectious compounds, there is compelling evidence that AMPs display antitumor activities in analogy to the antimicrobial, electrostatic interaction-based mechanism of action [102]. Thorough analysis of membrane activity of LL-37 and other AMPs revealed that membrane-permeabilizing agents affect mainly prokaryotic cells without considerable damaging eukaryotic cells. The most recognized theory assumes that this phenomenon is caused by a difference in the electrical charge characteristics of the cellular wall of these cells, and correlations may be drawn between membrane structures between bacteria and cancer cells (Fig. 2). Unlike bacterial cells, untransformed eukaryotic cell membranes expose anionic phospholipids primarily in the internal plasma membrane leaflet; thus, positively-charged chemotherapeutics are not highly attracted to the outer leaflet. In contrast, cancer cells are characterized by elevated negative charges on their cell surfaces, in part due to incorporation of phosphatidylserine in the outer leaflet of the plasma membrane [103]. These charges are enhanced by the presence of heparin sulfates [104], chondroitin sulfate [105] and O-glycosylated mucins [106] on their surfaces. Nevertheless, it should be noted that simple electrostatic interactions between AMPs and cell surfaces are insufficient for selective targeting of tumor cells since electronegative charges on the cell surface do not always increase AMP anti-cancer activity as seen with cell lines expressing and not expressing heparin sulfate on the cell surface [105]. Other cellular physiology-associated factors enhancing the selectivity of AMPs for cancer cells and facilitating killing are: (i) enhanced fluidity of cancerous cell membranes associated with incorporation of lower levels of cholesterol in transformed cells [107–109] (there are, however, conflicting reports [110, 111]) and (ii) increased surface area with enhanced number of microvilli, which facilitate the binding of AMPs and disruption of cell membranes [112]. In addition, multiple non-membranolytic mechanisms have been proposed including (i) intensification of apoptosis processes [113], (ii) disruption of mitochondria and thus, stimulation of the apoptotic cascade by caspase 3 activation [114], (iii) induction of T lymphocyte-mediated immune responses [115] and (iv) enhancement of the lysosomal-mitochondrial death pathway [116]. To date, a broad spectrum of AMPs, including α-defensins, BMAP-28 and cecropins, were reported to possess a potent, strongly-selective activity against cancer cells [102, 117]. Among this group, human cathelicidin-derived LL-37 peptide has drawn the attention of scientists due to its multifaceted biological activity, including antimicrobial, anti-cancer, immunomodulatory and wound healing properties [55].

LL-37 has been reported to exert variable and contradictory, tissue-specific impacts on cancer development, depending on the type of tissue from which cancer originates. These properties of LL-37 may be due to its ability to be a ligand for a broad spectrum of the membrane and intracellular molecular factors, whose expression varies considerably among cancer tissues [118]. A growing number of studies confirm that LL-37 increases the invasiveness and worsens the prognosis and clinical outcome in ovarian cancer [119–121], lung cancer [122], breast cancer [123], malignant melanoma [124] and prostate cancer [125] by mechanisms involving (i) recruitment of multipotent mesenchymal stromal/stem cells (MSCs) into the tumor stroma [126] and stimulation of EGFR, IGR-1R and ERB-family receptors [122, 123, 127]. Conversely, LL-37, its fragments and synthetic analogs have a great potential in cancer treatment [118]. Extensive research using cancer cell lines with clinical significance show that LL-37 acts as anti-cancer agent in gastric cancer [128, 129], colon cancer [130–132] and hematological malignancies [133] due to (i) activation of caspase-independent apoptosis [134], (ii) inhibition of angiogenesis [129], (iii) limiting proteasome activity [128] and (iv) controlling IL-32γ-induced inflammation [135]. These reports suggest that defining the understanding of this peptide on cancer development and its therapeutic potential is significantly hampered, due in part to variable expression of LL-37 in tumors when compared to healthy tissue of the same origin.

In relation to studies describing antimicrobial activities, fewer reports have been published describing the therapeutic value of LL-37 or cerageninn-containing nanosystems as anti-cancer agents. Nevertheless, promising data have been reported with colon [136] and breast cancer [137]. To date, detailed investigation of the anti-tumor mechanism of LL-37 action in colon cancer has shown that human cathelicidin-derived peptide (similarly to its modified versions—FK-16 [i.e. 16-residue peptide derived from residues 17–32 of LL-37 [138] ] and FF/CAP [designed by replacement of glutamic acid and lysine residue with phenylalanine]) regulates cancer development by increase of PUMA expression (i.e. direct target for p53 and a modulator of apoptosis process) followed by induction of caspase-independent apoptosis and autophagy through the p53-Bcl-2/Bax signaling pathway [130–132] or p53-indepenent mechanism [132]. In recent studies, human cathelicidin was reported to interfere with tumor growth factor-β1-induced epithelial–mesenchymal transition (EMT) of colon cancer cells and fibroblast-supported colon cancer cell proliferation [139], which highlights that the spectrum of anti-tumoral activities of this peptide. Interestingly, in colon cancer HCT116 cells treated with LL-37 or FF/CAP upregulation of miR-663a (i.e. miRNA associated with anti-proliferative effects) was noted, which is determined by inhibited expression of CXCR4 receptor and cell cycle arrest in G2/M via p21 activation [140]. Additionally, a study conducted by Kuroda et al. revealed that ceragenin CSA-13 also possesses significant anti-cancer activity, and in this study it was concluded that CSA-13 decreases the survival of colon cancer cells via induction of apoptosis processes [141].

Research by Niemirowicz et al. indicates that, in analogy to nanosystems possessing high antimicrobial activity, magnetic nanoparticles intensify the anti-cancer effects of LL-37 and ceragenin CSA-13, as evaluated using colon cancer DLD-1 and HT-20 cells [136]. When comparing to free molecules, LL-37 and CSA-13 attached to the surface of iron oxide-based magnetic nanoparticles with an aminosilane shell (MNP@LL-37 and MNP@CSA-13, respectively) have a greater ability to decrease cell viability and induce apoptosis, which is correlated with effective internalization of these molecules into the nucleus of HT-29 cancer cells. These observations suggest that employment of AMPs and ceragenins as novel homing molecules may allow development of targeted therapy due to their ability to accumulate in the nucleus. The exact mechanism of this phenomenon is still unclear; nevertheless, it is assumed that LL-37 attached to the surface of MNPs is able to better activate anti-tumorigenic pathways, stimulated or inhibited by its unmodified form. Moreover, reports indicating enhanced cellular uptake efficiency mediated by aminosilane surface coatings [142] strengthen the hypothesis of improved delivery of membrane-active compounds into cancer cells and of synergistic effects of MNPs and cationic antimicrobial peptides [136].

Similar improvement of anti-cancer activity of CSA-13, through generation of MNP, was reported for ceragenin-mediated breast cancer treatment. Recently, Piktel et al. reported that ceragenin CSA-13 exerts significant anti-cancer activity against breast cancer MCF-7 and MDA-MB-231 cells lines, which results from a novel mechanism involving cell membrane disorganization and induction of caspase-dependent apoptosis via increased reactive oxygen species (ROS) generation followed by mitochondrial membrane depolarization [137]. This oxidative balance disruption-mediated effect was noted to be further strengthen due to attachment of CSA-13 to the surface of MNPs, leading to improvement of cellular internalization of membrane-active compounds into breast cancer cells and most likely, intensification of oxidative stress due to ROS-generating abilities of uncoated nanoparticles [143]. This uptake results in damage to cellular proteins, disruption of mitochondrial membrane polarization and cell death [137]. One of the novel nano-based approaches tested to date assumed that attachment of LL-37, as a compound with variable receptor-dependent activity against cancer cells and considerable membrane-permeabilizing properties [118], on the surface of magnetic nanoparticles characterized by well-described anti-cancer activity [144] would provide means of reversing pro-tumorigenic activity of the human cathelicidin and allow for creation of a nanosystem with anti-cancer features and high biocompatibility. This hypothesis was strengthened by previous reports of increases in anti-neoplastic effects of CpG oligodeoxynucleotides against ovarian cancer in the presence of LL-37, despite the pro-tumorigenic activities of human cathelicidin in ovarian tissues [145]. Unfortunately, despite the partial reverse of negative effects of LL-37, anti-cancer activity of such combination was weak, as compared to strong, anti-neoplastic activity of CSA-13 and MNP@CSA-13 [137].

**Fig. 3:**
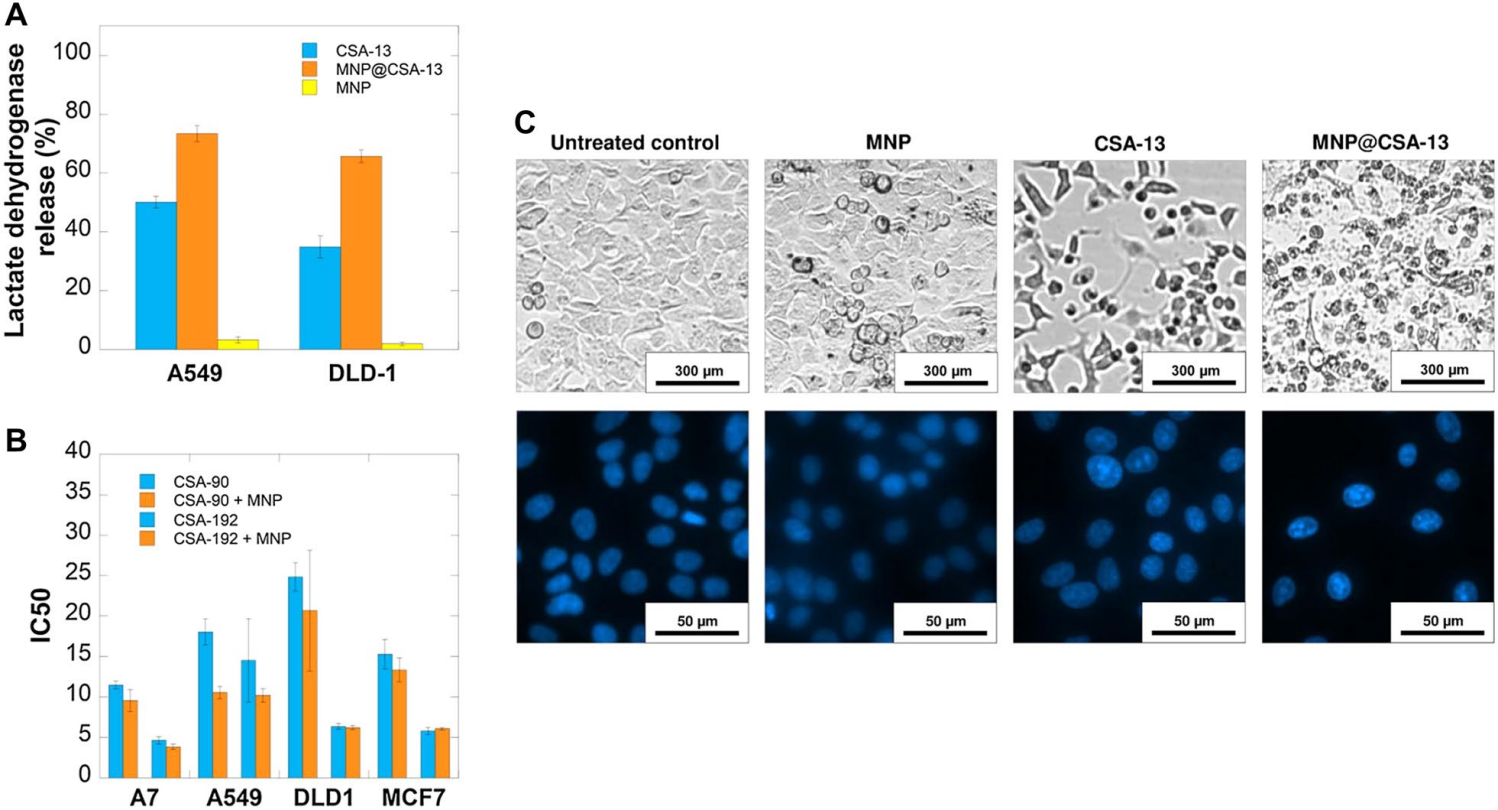
Improvement of anti-cancer activities of ceragenins CSA13, CSA-90 and CSA-192 after their immobilization on the surface of magnetic nanocarriers. **a** The level of lactate dehydrogenase release from lung carcinoma A549 and colon cancer DLD-1 cells after treatment with 10 µg/mL of ceragenin CSA-13 and its magnetic derivative. **b** IC50 values recorded for CSA-90, CSA-192 and their mixture with MNP against malignant melanoma A7, lung carcinoma A549, colon cancer DLD-1 and breast cancer MCF-7 cells. **c** Alterations in morphology and DNA fragmentation in colon cancer DLD-1 cells after their treatment with CSA-13 and MNP@CSA-13 at dose of 10 µg/mL for 24 h, investigated using phase contrast and fluorescence microscope, respectively

In addition to these reports, our unpublished data confirm that the spectrum of anti-neoplastic activity of ceragenins and ceragenin-based magnetic nanosystems is not limited to colon and breast cancer. Analyzes of anti-cancer activities of ceragenins CSA-13, CSA-131, CSA-90 and CSA-192, both in free form and immobilized on the surface of core–shell magnetic nanoparticles reveal their therapeutic value in the treatment of lung carcinoma, colon cancer, breast cancer and malignant melanoma. Importantly, the increase of anti-neoplastic activities of membrane active compounds might be achieved by both covalent immobilization or electrostatic-based bond between ceragenins and magnetic nanocarriers (Fig. 3).

## Multifaceted activities of nanosystems based on membrane active compounds—future directions and possible applications

### Nanoantibiotics as novel immunomodulatory compounds

The promising results obtained using drug-resistant microbial pathogens and cancer cells demonstrate the multifaceted and pleiotropic activities of human cathelicidin and its lipid analogs, ceragenins. These activities are in addition to the immunomodulatory and regenerative activities of nanosystems consisting of LL-37 and ceragenins. LL-37 possesses LPS- and LTA-binding ability and thus, limits the prostimulatory effects of bacteria-derived molecules, as evaluated using CD14+ murine macrophage cell line (RAW264.7) and the murine endotoxin-shock model. Accordingly, LL-37 was reported (i) to inhibit the binding of LPS to macrophages [146], (ii) suppress LPS-induced tumor necrosis factor (TNF)-α mRNA and protein expression [146], (iii) prevent the binding of LPS to LPS-binding protein and its receptor [147] and (iv) limit the expression of LPS-induced genes in endotoxin-stimulated monocytes [148]. More importantly, it is established that LL-37 exerts pleiotropic activities due to interaction with a broad spectrum of membrane receptors, including P2X7 [149] and formyl peptide receptor-like 1 (FLPR-1), which allow for modulation of the inflammatory response [55, 150]. Considering that ceragenins, as lipid analogs of antimicrobial peptides, mimic a variety of AMP’s features, including amphipathic chemical character, antimicrobial and anti-cancer activities, it is assumed that also other AMP’s characteristics (e.g. promotion of cell migration, neovascularization, regenerative and immunomodulatory properties) will be displayed among this group. Indeed, despite the considerably lower number of studies aiming to evaluate these features in ceragenins, it has been shown that ceragenins suppress the immunostimulatory effect of bacterial wall components (i.e. LPS and LTA) since they are highly capable of sequestering bacterial endotoxins and in result, limit the immune system responses. CSA13-mediated binding of LPS and LTA from multiple types of bacteria, inhibition of TLR4-mediated NF-κB translocation to the nucleus and limitation of proinflammatory cytokines release [20, 151] have been reported. Mimicking of LL-37-associated effects by ceragenins was also highlighted by one of recent studies demonstrating protective role of this agent in colitis-associated interstitial fibrosis, which is determined by activation of FPRL-1, and thus reduction of fibroblast accumulation and exerting of anti-fibrogenic activity [152]. In addition, nanomaterials, including magnetic nanoparticles, were reported to interact with immune cells and either stimulate or suppress immune responses and particle specific features such as size, shape or surface chemistry were shown to control the type of response [153–155].

Multiple in vitro and in vivo studies have been reported aiming to assess the impact of iron oxide-based magnetic nanoparticles on immune response. Most recently, magnetic nanoparticles were noted to modulate LPS-induced inflammatory responses in primary human monocytes and suppress inflammatory responses in mice models when administrated both intravenously [156] or intratracheally [157]. In analogy to antimicrobial and anti-cancer nanoformulations, aminosilane- (MNP@NH_2_) and gold-coated magnetic nanoparticles (MNP@Au) were also noted to exert anti-inflammatory effects in LPS/LTA-stimulated keratinocytes and augment the beneficial effects of PBP10 peptides, which are derived from human plasma gelsolin (pGSN) and are structurally and functionally similar to AMPs (cationic charge, a short sequence, membrane-permeabilizing properties and amphipathic chemical character and resulted from this, broad spectrum of antimicrobial activity) [158]. Considering these studies, it might be assumed that LL37/ceragenin-containing nanosystems might improve immunomodulatory functions relative to the parent compounds as a result of the combined anti-inflammatory effects of AMPs and magnetic nanoparticles. Generation of AMPs or ceragenin-based NPs may offer a novel approach for the development of improved anti-infectious agents that additionally have anti-inflammatory functions.

### The employment of ceragenin-containing nanosystems in regenerative medicine

Some studies suggest that nanosystems containing membrane active compounds, in addition to their high antimicrobial, anti-cancer and immunomodulatory potential should be studied in the context of regenerative medicine. Although the amount of research elucidating the regenerative features of ceragenins is still limited, there are some data about the improvement of human osteoblasts hFOB1.18 cell proliferation in the presence of relatively low doses of ceragenin CSA-13 and its magnetic derivative, MNP@CSA-13. As demonstrated by Niemirowicz et al., 24 h-incubation of osteoblasts cells with LL-37, CSA-13 and their magnetic-based nanosystems does not affect significantly the proliferation process, while stimulating their growth at concentrations ranging from 5 to 10 µg/mL. Importantly, this effect was more prominent for functionalized nanoagents when compared to free compounds, and ceragenin-mediated treatment exerted higher impacts than natural AMPs [84]. Some ceragenin compounds, including CSA-13, CSA-90, CSA-142 and CSA-192 have been also shown to stimulate human keratinocytes (HaCaT) migration in low concentration by mechanisms involving induction of angiogenesis processes (as evaluated using in vitro tube formation assay) via vascular endothelial growth factor receptor 2 (VEGFR2)-dependent pathway. Moreover, CSA-90 was reported to promote osteogenesis, enhance matrix mineralization in cultured osteoblasts and increase rhBMP-2-induced bone formation in an in vivo rat open fracture model of *S. aureus* infection, which represents a novel therapeutic approach with specific benefits in the context of orthopaedic injuries [159]. Considering the non-toxic effect of low doses of ceragenins on mammalian cells, followed by regenerative potential of magnetic nanoparticles, it might be assumed that antimicrobial peptides-containing nanoantibiotics in the future will be translated into innovative tools characterized not only by broad spectrum of antimicrobial activity, but also by anti-inflammatory properties and regenerative features, which may be crucial in the context of delayed wound healing of infected tissues.

### AMP-based nanosystems for development of next-generation implantable medical devices

Due to ever-growing public health and economic burden related with device-associated hospital infections, increased efforts have been made to develop next generation abiotic surfaces and surgically implantable medical devices on which microorganisms have limited ability to adhere and form biofilms. Considering that bacteria and fungi embedded with biofilms are nearly completely resistant to conventional antibiotics, it is indisputable that biofilms formed on medical devices (particularly those with long resident times) are a continuous source from which pathogens are released to surrounding tissues causing serious health complications and life-threating infections. Traditional antibiofilm approaches, including physical deposition of antimicrobials, surface oxidation/reduction or coating with copolymer have been insufficient [160]. Therefore, alternative methods of preventing surfaces colonization with biofilm are required.

One of the newer approaches assumes the coating of medical devices and implants with bare NP or NP-based formulations. Particularly, silver NP and silver-based conjugates are currently proposed for coating of medical devices due to their potent antimicrobial activity [161, 162]. In addition, application zinc oxide, iron oxide, titanium dioxide and copper oxide-based NP has also been explored [163].

The ceragenins and ceragenin-based nanoformulations appear well suited for preventing biofilm formation on medical devices. Pollard et al. suggested the employment of ceragenin CSA-13 as suitable factor eliminating established biofilms of both Gram-positive and Gram-negative bacteria at concentrations comparable to ciprofloxacin but retaining killing capabilities against drug-resistant pathogens [164]. More recently, ceragenins were incorporated into contact lenses or used as a coating on fracture fixation plates for the purpose of limitation of *P. aeruginosa* and *S. aureus* biofilm prevention and eradication [165, 166]. These results were due to the potent antimicrobial and antibiofilm activities of CSA-138 and CSA-13, high stability in the presence of body fluids and favorable thermal, chemical and physical properties in pre-polymers solutions. Most importantly, the value of these agents as coating anti-biofilm factors were confirmed in in vivo models, indicating not only successive protection of CSA13-coated plates from MRSA-induced infection but also demonstrating good tolerance of this approach. Most recently, report by Hashemi et al. has shown that thin films containing CSA-131 protect endotracheal tubes against microbial colonization decreasing adverse effects of intubation associated with infection and inflammation [167]. Currently, there is an urgent need to develop novel antibiofilm coatings for implantable cardiac devices, including cardiac resynchronization devices (CRT) or pacemakers (PM), which are commonly colonized by *S. aureus* or fungi and introduced during implantation surgery. Importantly, cardiac device infections can result in a broad range of complications from wound infections and pacemaker infective endocarditis [168]. Combining the antimicrobial effects of NPs and ceragenins may be an effective strategy to improve antibiofilm efficiency of these compounds in coatings, which will directly contribute to improvement of medical devices and lower microbial contamination.

## Conclusions

Rapid development in the field of nanotechnology-based therapeutic approaches has resulted in the design of novel and innovative nanodrugs with special characteristics, suitable for the treatment of life-threating medical conditions, including drug-resistant microbial infections and cancers. Unique physicochemical features of magnetic NPs make them interesting tools for development of modern, highly efficient therapeutic nanoagents. The employment of iron oxide-based NPs as drug delivery systems seems to have the biggest impact currently; nevertheless, the ever-growing number of studies describing the therapeutic values of magnetic NPs and nanoantibiotics in the context of magnetic fluid hyperthermia inductors and enhancers of photosensitizers highlight their strong potential for therapeutic use. Surface immobilization of AMPs such as human cathelicidin-derived LL-37 peptide and its synthetic analogs, ceragenins, has attracted attention. Studies described in this review confirm or strongly suggested the possibility of development AMP and ceragenin-based magnetic nanosystems as novel and innovative tools with broad spectrum of antimicrobial features, anti-cancer activity and immunomodulatory and regenerative potential.

## Data Availability

Materials described in the manuscript, including all relevant raw data, will be freely available to any scientist wishing to use them for non-commercial purposes upon request via e-mail with corresponding author.

## Abbreviations

AMPs: antimicrobial peptides
CAPs: cationic antimicrobial peptides
CSA: cationic steroid antimicrobials, ceragenins
EMT: epithelial–mesenchymal transition
FF/CAP: LL-37 derivative designed by replacement of glutamic acid and lysine residue with phenylalanine
FK-16: 16-residue peptide derived from residues 17–32 of LL-37
FPRL-1: formyl peptide receptor-like 1
IL: interleukin
LL-37: human cathelicidin-derived LL-37 peptide
LPS: lipopolysaccharide
LTA: lipoteichoic acid
MNP: magnetic nanoparticles
MNP@Au: gold-coated magnetic nanoparticles
MNP@CSA: ceragenin immobilized on the surface of magnetic nanoparticles
MNP@LL-37: human cathelicidin-derived LL-37 peptide immobilized on the surface of magnetic nanoparticles
MNP@NH2: aminosilane-coated magnetic nanoparticles
NPs: nanoparticles
PAE: post-antibiotic effect
ROS: reactive oxygen species
TLR: toll-like receptor
TNF-α: tumor necrosis factor alpha
